# Age-Dependent Paraoxonase 1 (PON1) Activity and LDL Oxidation in Wistar Rats during Their Entire Lifespan

**DOI:** 10.1155/2014/538049

**Published:** 2014-05-22

**Authors:** Dileep Kumar, Syed Ibrahim Rizvi

**Affiliations:** Department of Biochemistry, University of Allahabad, Allahabad, Uttar Pradesh 211002, India

## Abstract

Paraoxonase 1 (PON1) is an HDL bound enzyme which plays a key role in the protection of LDL and HDL from oxidation by hydrolyzing activated phospholipids and lipid peroxide products. Oxidative stress plays a crucial role in the development of atherosclerosis by oxidation of LDL. This study was conducted to determine age-dependent changes in plasma PON1 arylesterase activity and LDL oxidation in rats during their entire lifespan. 48 Wistar strain rats were grouped in six different age groups (1, 4, 8, 12, 18, and 24 months). We observe a significant (*P* < 0.001) age-dependent decrease in plasma PON1 arylesterase activity correlating with increase in susceptibility of LDL oxidation and increase in plasma MDA level concomitantly with a significant (*P* < 0.001) decrease in plasma radical scavenging activity after 8 months. The reduction of PON1 and free radical scavenging activity with age could have a considerable impact on the increased incidence of atherosclerosis with age. Our observation of a significant decline in PON1 activity which correlates with increased LDL oxidation after 8 months of age is an interesting observation and needs further investigation.

## 1. Introduction

Aging is considered a gradual, time-dependent accumulation of molecular damage leading to lower functionality and resistance. The most important cause of molecular damage has been implicated to be free radical mediated oxidative stress which may be due to extrinsic or intrinsic factors [1]. The free radical theory of aging provides an attractive mechanistic approach to explain molecular changes in DNA, lipids, and proteins associated with the aging process [2]. Lipid peroxidation is a well-established mechanism of cellular injury and is used as an indicator of oxidative stress in cells and tissues [3]. Elevated lipid peroxide levels with aging have been demonstrated in various tissues and cells including the liver, brain, kidney, heart, and erythrocytes [4, 5]. Increased levels of lipid peroxidation products have been associated with a variety of diseases in both humans and model animal systems [3].

Oxidation of LDL is a lipid peroxidation process in which the polyunsaturated fatty acids (PUFA) in the LDL are first converted to lipid hydroperoxides and subsequently to aldehydic lipid peroxidation products [6]. Oxidative stress plays a crucial role in the development of atherosclerosis by oxidation of LDL which leads to formation of foam cells [7]. Conversely, high-density lipoprotein (HDL) is a well-known antioxidant molecule that prevents atherosclerosis [8]. Paraoxonase 1 (PON1) is an HDL bound enzyme which plays a key role in the protection of LDL and HDL from oxidation by hydrolyzing activated phospholipids and lipid peroxide products [9]. PON1 activity is reduced during cardiovascular diseases and cancer [10], as well as during acute infections [11]. Previous researches show that both LDL and HDL have an increased susceptibility to oxidation with age [12]. In a recent study we have shown a decrease in PON1 arylesterase activity in humans during aging which shows a correlation with the susceptibility of LDL oxidation [13].

In the present study we have investigated the PON1 arylesterase activity and susceptibility of low-density lipoproteins for induced oxidation as a function of age in rats during their entire lifespan (24 months). We have also determined the antioxidant potential of plasma during aging along with plasma lipid peroxidation and we have made an effort to establish correlations between all these parameters.

## 2. Materials and Methods

### 2.1. Chemicals

Phenyl acetate, copper chloride, and DPPH were purchased from Sigma Aldrich, India. All other chemicals were of highest purity available from Merck, India, and HIMEDIA Labs, India.

### 2.2. Animal Model and Study Protocol

The experiment was carried out on 48 male Wistar rats. They were housed in a temperature controlled room (25 ± 5°C) with 12 h light-dark cycles. All rats were fed with normal laboratory diet in the form of nutrient rich pellets containing total energy as fat, protein, and carbohydrates, with free access to drinking water. The rats were divided into six groups based on ages corresponding to 1, 4, 8, 12, 18, and 24 months. A randomly selected group of animals of each age were sacrificed under light anesthesia to obtain fresh venous blood.

### 2.3. Collection of Blood and Isolation of Red Blood Cells and Plasma

Blood samples were collected by cardiac puncture into 10 unit/mL heparin and then red blood cells were pelleted by centrifugation at 800 g for 10 min at 4°C. The plasma was immediately frozen at −80°C for biochemical assays. The protocol of study was approved by the Animal Care and Ethics Committee of University of Allahabad.

### 2.4. Determination of PON1 Arylesterase Activity

This assay was performed by the method developed by Ayub et al. [14]. Enzyme activity toward phenyl acetate (arylesterase activity) was determined by measuring the initial rate of substrate hydrolysis in the assay mixture (3 mL) containing 2 mM substrate (phenyl acetate), 2 mM CaCl_2_, and 10 *μ*l plasma in 100 mM Tris-HCl (pH 8.0). The absorbance was monitored for 3 min at 270 nm and the activity was calculated from E270 = 1310 per M/cm. The results are expressed in U/mL, 1 U of arylesterase hydrolyses 1 mmol of phenyl acetate per minute.

### 2.5. Measurement of LDL Oxidation

This assay was performed according to the method developed by Schnitzer et al. [15]. Rate of LDL oxidation was measured in assay mixture (2 mL) containing 0.72 mM sodium citrate, 90 *μ*M copper chloride and 40 *μ*l plasma in 10 mM phosphate buffer (pH 7.4). Absorbance was monitored at 245 nm for 3000 seconds and graph was plotted for absorbance versus time. Age-dependent LDL oxidation was obtained by measuring oxidation at 3000 seconds.

### 2.6. Radical Scavenging Activity of Plasma

This assay was performed according to the method proposed by Szabo et al. [16]. 100 *μ*l of plasma was added to 10 mM phosphate buffer (1.9 mL) and 0.1 mM 2,2-diphenyl-1-picrylhydrazyl (DPPH) in methanol (2.0 mL) with a control having 2 mL of 10 mM phosphate buffer with the same amount of DPPH solution. It was kept for incubation for 30 min at 21°C and centrifuged for 5 min at 1000 ×g. Absorbance was measured at 517 nm with methanol as a blank. Values were compared for control (A0) and plasma (A) and percent radical scavenging activity (%RSA) was calculated by using 100 (A0 − A)/A0.

### 2.7. Determination of Plasma Lipid Peroxidation

Plasma lipid peroxidation in terms of malondialdehyde (MDA) was measured according to the method of Esterbauer and Cheeseman [17], with slight modification. Plasma (0.2 mL) was added to 1 mL of 10% trichloroacetic acid (TCA) and 2 mL of 0.67% thiobarbituric acid (TBA) boiled for 20 min at 90–100°C and cooled; the mixture was centrifuged at 1000 g for 5 min and the absorbance of supernatant was read at 532 nm. The concentration of MDA in plasma was calculated using extinction coefficient (*ε* = 31,500) and is expressed as nmol · mL^−1^ of plasma.

### 2.8. Statistical Analysis

All data are presented as means ± SEM. Statistical analyses were conducted using the software PRISM version 5.01.* t-*test was used to assess relationships between parameters and differences among treatments. Multiple comparisons were done by employing one way analysis of variance (ANOVA) and subjecting Bonferroni posttest on the data.

## 3. Results

**Figure 1:**
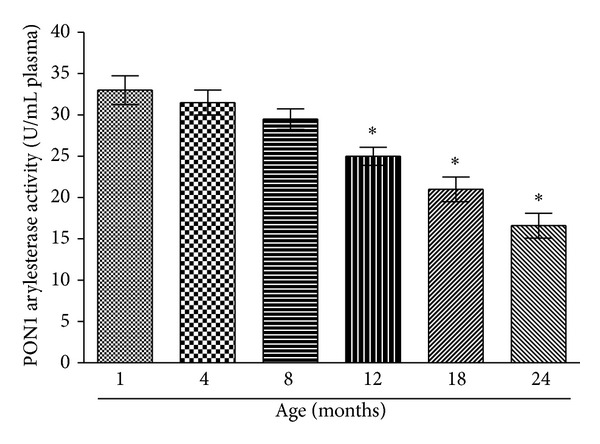
Plasma paraoxonase 1 (PON1) arylesterase activity as a function of rat age. PON1 activity is expressed as U/mL plasma. Values are mean ± SEM. *P < 0.001 compared to control (1-month-old rat).

Our results show decrease in PON1 arylesterase activity as a function of rat age (Figure 1). The decrease is not significant till 8 months of age; however at 12 months of age the decrease in PON1 activity is significant (*P* < 0.001) compared to the control value which in our experimental conditions has been taken at 1 month.

**Figure 2:**
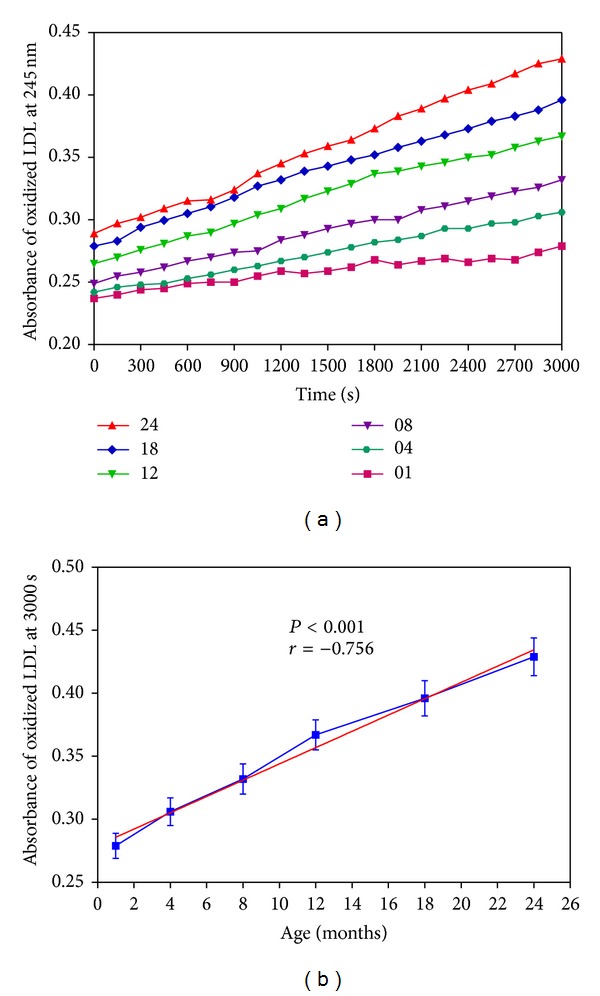
(a) Absorbance at 245 nm as a function of time for induced LDL oxidation in plasma of different age group rats (age in months). (b) The absorbance of oxidized LDL samples at 3000 seconds as a function of rat age (in months). Values are mean ± SEM.

Susceptibility of plasma LDL oxidation towards induced oxidation increased with age in rats.  Figure 2(a) shows the kinetics of the LDL oxidation as a function of time for selected samples representing different ages; the highest rate of LDL oxidation is observed in 24-month-old rat plasma.  Figure 2(b) shows the graph of LDL oxidation (values derived after 3000 sec) as a function of rat age. A significantly (*P* < 0.0001) higher oxidation of LDL is observed in old age rats (24 months) compared to young age rats (4–8 months).

**Figure 3:**
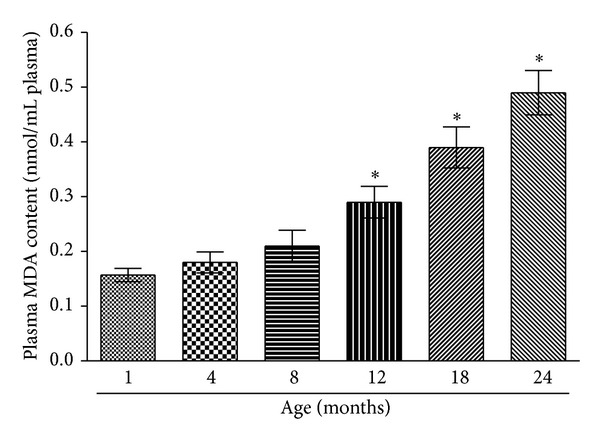
Plasma malondialdehyde (MDA) content as a function of age of rats (in months). MDA level is expressed as nmol · mL^−1^ of plasma. Values are mean ± SEM. *P < 0.001 compared to control (1-month-old rat).

**Figure 4:**
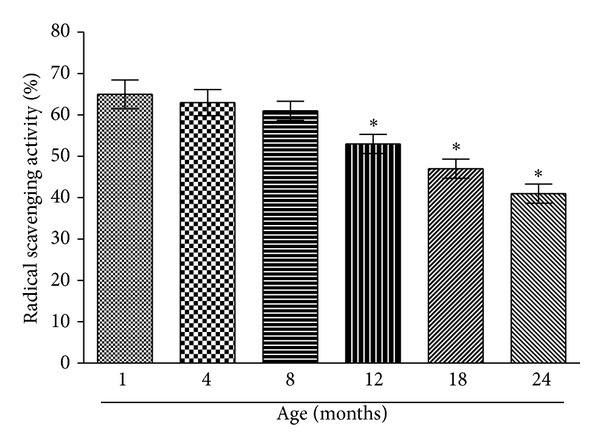
DPPH radical scavenging activity of plasma plotted as a function of rat age. Values are mean ± SEM. *P < 0.001 compared to control (1-month-old rat).

Plasma lipid peroxidation in the study groups is given in Figure 3. Plasma MDA levels increase at a constant rate from 1 month to 8 months of age; however, there is a sudden increase in lipid peroxidation in 12-month-old rats which is significantly (*P* < 0.001) higher compared with 8-month-old rats. Our result shows that radical scavenging activity of plasma towards DPPH decreases as a function of age (Figure 4). Radical scavenging activity of plasma does not significantly decrease till 8-month age of rats; however, it significantly (*P* < 0.001) decreases after 8 months of age.

**Figure 5:**
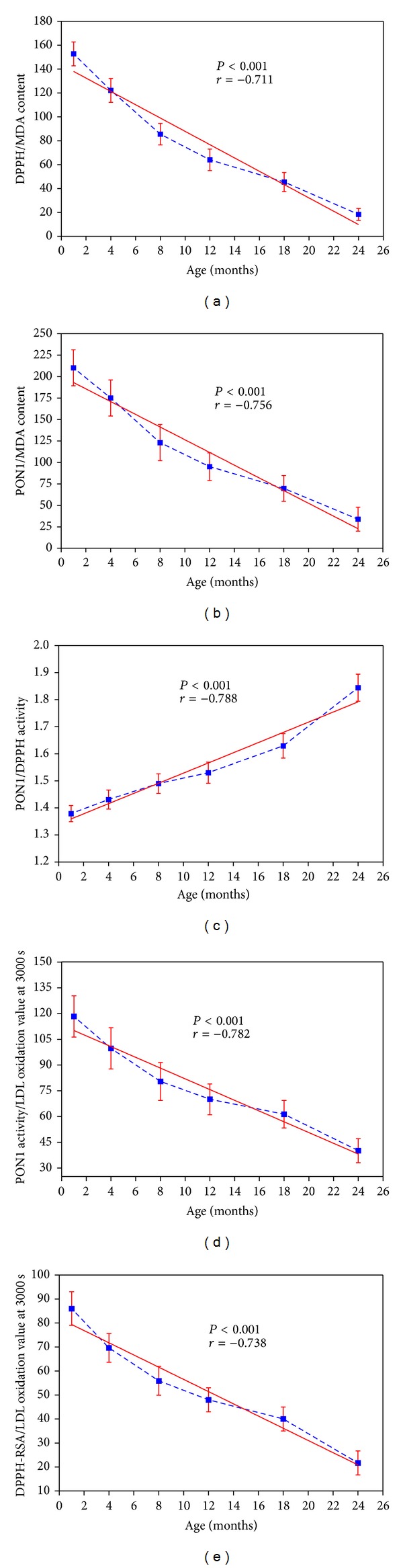
(a) Correlation of quotient of DPPH-RSA and plasma MDA level as a function of rat age. (b) Correlation of quotient of PON1 arylesterase activity and MDA level as a function of rat age. (c) Correlation of quotient of PON1 arylesterase activity with plasma DPPH-radical scavenging activity as a function of rat age. (d) Correlation of quotient of PON1 arylesterase activity and LDL oxidation level at 3000 sec plotted as a function of rat age. (e) Correlation of quotient of DPPH-RSA and LDL oxidation level at 3000 sec plotted as a function of rat age.

Figure 5(a) shows the correlations of ratio DPPH/radical scavenging activity with MDA (Pearson's *r* = −0.711 and *P* < 0.001) and Figure 5(b) gives the correlation between PON1 activity and MDA value (Pearson's *r* = −0.756 and *P* < 0.001).  Figure 5(c) shows a strong positive correlation between PON1 and DPPH radical scavenging activity (Pearson's *r* = 0.788 and *P* < 0.001).  Figure 5(d) exhibits the correlation between PON1 arylesterase activity and oxidized LDL (Pearson's *r* = −0.782 and *P* < 0.001) and correlation of DPPH-RSA with LDL oxidation value at 3000 sec as a function of age (Pearson's *r* = −0.738 and *P* < 0.001) is shown in Figure 5(e).

## 4. Discussion

The existence and the role of increased extra- and intracellular oxidative stress during aging and aged-related disorders are an area of interest [2, 18]. The antioxidant roles of PON1 are well established and supported by clinical studies [19]. Decreased PON1 activity has been reported in various diseases such as diabetes mellitus, atherosclerotic heart disease, rheumatoid arthritis, and chronic renal failure [10]. Interestingly, the proteomic analysis of plasma proteins in semisuper centenarians also shows decrease in the paraoxonase arylesterase activity [20]. It is well documented that PON1 deficiency is related to increased susceptibility for low-density lipoprotein oxidation and development of atherosclerosis [8, 21].

Oxidative imbalance has been implicated in the etiology of various disorders, including cancers, renal diseases, Parkinson's disease, Alzheimer disease, liver disorder, and diabetes mellitus [10]. In an earlier study, Seres et al. [22] demonstrated a decrease in PON1 activity with aging. Serum PON1 activity has also been shown to be modulated by lifestyle and dietary factors such as short-term caloric restriction [23]. Seres et al. [22] suggested that increasing oxidative stress with aging could explain, in part, the observed reduction in PON1 activity of the elderly. PON1 not only affects the process of cholesterol efflux via an ABCA1 dependent pathway [24], but also shows an antioxidative role in familial hypercholesterolemia [25]; so its activity is an adjunctive index of altered lipoprotein metabolism [26]. Therefore the reduction in the PON1 activity could negatively affect age-dependent diseases and also the rate of aging process.

The role of free radical-induced lipid oxidation during aging and development of oxidative stress has been reported earlier and it has been suggested that the degree of lipid peroxidation correlates with free radical progression [4, 5]. We estimated MDA levels in rat plasma during aging along with PON1 enzymatic activities. Our results are in agreement with earlier studies [27]; we observed significantly elevated levels of MDA during aging in rats. Furthermore, we observed a negative correlation between quotient of paraoxonase activity and MDA levels as a function of age. The inverse relationship between lipid peroxidation and PON1 activity is not unexpected as PON1 activity is known to be inhibited by oxidized LDL [9].

Decrease in serum PON1 activity under oxidative stress has been mostly attributed to changes in the redox status of the protein's free sulfhydryl group which prevents the inhibition of PON1 activity caused by reactive oxygen species [28]. Free sulfhydryl groups of proteins constitute the main antioxidant component of serum. Also there is some evidence from animal models that PON1 can protect the HDL particle from oxidation and preserve the integrity of HDL [12]. Our observation of a positive correlation between PON1 activity and radical scavenging activity of plasma provides an explanation for age-dependent decline in PON1 activity.

Low-density lipoproteins (LDL) have been found to be prone to oxidation with increasing age. Under oxidative stress, oxidative modification of LDL takes place in the subendothelial space of the arterial wall and a certain amount of oxLDL is released into the circulation [29]. LDL oxidation affects both the protein and the lipid content, resulting in the formation of lipid peroxidation metabolites [30]. OxLDL, apart from being a biomarker of oxidative status, has also been reported to be a potent independent mitogenic factor [31]. Our study suggests that the increase in the susceptibility of low-density lipoprotein for oxidation is due to the decrease in serum PON1 arylesterase activity with age.

In a recent report on humans we reported a continuous decrease in plasma PON1 arylesterase activity starting from 20 years of age till 80 years [13]. It is of interest that in rats there is no significant change in PON1 arylesterase activity, plasma MDA, and radical scavenging activity till 8 weeks of age which is almost one-third of the total lifespan of 24 months. Since the activity of PON1 depends on many factors including genetics [32], it is possible that subtle changes in its activity in humans get masked due to difficulties in maintaining standard conditions; however, rats can be bred and kept in comparatively standard conditions and hence such changes in enzyme activity are measurable. Our observations emphasize the existence of an effective antioxidant defense in rats during the early part of their lifespan. To the best of our knowledge, this is the first evidence of an interaction between PON1 activity and LDL susceptibility to oxidative changes during aging in rats.

## 5. Conclusion

On the basis of our present observations and in view of recent reports, we show a significant decrease in PON1 activity with age which may be due to the increased susceptibility of LDL oxidation and elevated oxidative stress during aging. We provide evidence for the increase in plasma lipid peroxidation level with age and reduction in plasma radical scavenging activity. The relationship of PON1 status to CHD is closely related and prospective studies confirm that PON1 is an independent risk factor for CHD [33]. Thus the reduction of PON1 and free radical scavenging activity with age could have a considerable impact on the increased incidence of atherosclerosis with age. Further studies are needed to clarify the mechanism of the decrease in PON1 activity in rats and also to understand the underlying defense systems which operate to protect rats from oxidative changes till 8 months of age.
